# A pilot study of remote cognitive assessment in children using the NIH toolbox participant/examiner app

**DOI:** 10.1038/s41598-025-20256-7

**Published:** 2025-10-16

**Authors:** Berivan Ece, Emily H. Ho, Zutima Tuladhar, Miriam A. Novack, Shaili Ganatra, Anne Zola, Vitali Ustsinovich, Christine W. Hockett, Richard Gershon

**Affiliations:** 1 Department of Medical Social Sciences, Feinberg School of Medicine, Northwestern University, 625 N. Michigan Ave., 27th floor, Room 2745, Chicago, IL 60611 USA; 2 Kellogg School of Management, Northwestern University, Evanston, IL USA; 3 Avera Research Institute, Sioux Falls, SD USA; 4 Department of Pediatrics, University of South Dakota School of Medicine, Sioux Falls, SD USA

**Keywords:** Remote assessment, Remote cognitive assessment, NIH toolbox, NIH toolbox cognition battery, NIH toolbox participant/Examiner app, Neuroscience, Psychology

## Abstract

The demand for remote assessment tools has increased, yet there is a lack of standardized adaptations for remote administration. This pilot study investigates the equivalency of in-person and remote cognitive assessments using the NIH Toolbox Cognition Battery (NIHTB-CB) among children aged 7 to 17 years. Forty-seven children (51.1% female; *M*_*age*_ = 12.26, *SD*_*age*_= 3.23) were assessed in two formats: in-person at a study site and remotely from home, with the order of assessments counterbalanced. The NIHTB-CB was used for in-person evaluations, while a newly developed version, the *NIH Toolbox Participant/Examiner (NIHTB-P/E) App*, was used for remote assessments through built-in teleconferencing features. The results showed considerable consistency between in-person and remote scores across all NIHTB-CB tests. Certain differences were noted, including longer test durations for remote assessments and potential practice effects on some measures. Overall, preliminary findings from this pilot study support thefeasibility of administering the NIHTB-CB remotely using the NIHTB-P/E App, providing a viable option to traditional in-person cognitive assessments in pediatric populations.

**Supplementary Information:**

The online version contains supplementary material available at 10.1038/s41598-025-20256-7.

## Introduction

 Cognitive assessment is vital to evaluating cognitive functioning^1^. It is particularly critical during childhood due to its essential role in tracking healthy development, identifying any developmental delays, making accurate and timely diagnoses, and evaluating the effectiveness of treatments and interventions^2–6^. In that respect, cognitive assessments have significant implications for children, ranging from treatment decisions to eligibility for access to resources^3^. Additionally, cognitive abilities have been consistently associated with real-world outcomes, such as social functioning^7^ and physical and mental health^8^as well as educational outcomes including school readiness, early academic skills, language comprehension, reading, vocabulary acquisition, and mathematics^9–11^. Cognitive assessments are also used in research studies to compare outcomes between intervention groups. Therefore, it is crucial to have reliable, valid, and age-appropriate standardized measures to evaluate children’s cognitive functioning, as these tools ensure the quality and accuracy of the assessment process.

Traditional methods of cognitive assessment in pediatric populations often involve direct interaction between an examiner and a participant, typically conducted in a clinical, research, or educational setting using standardized paper-and-pencil or computerized tests^12–14^. The COVID-19 pandemic, however, interrupted these traditional in-person approaches due to social distancing requirements and restrictions on face-to-face interaction^15–17^ and led researchers to explore alternative assessment strategies. Remote assessment, in which the participant and examiner are in separate locations, has proven to offer several benefits to both researchers and participants. Benefits for researchers include increased efficiency of data collection, increased sample size and diversity, higher generalizability and ecological validity, and cost-effectiveness^18,19^. Benefits for participants include eliminating barriers to participation, such as travel costs and long travel hours, which is particularly helpful for participants residing in remote or hard-to-reach areas^20,21^. Remote testing can reduce attrition in longitudinal studies by increasing the likelihood of participation at multiple time points^21^. Remote assessment can even aid the recruitment of participants living with limited mobility (e.g., physical disabilities) who are underserved when in-person assessment strategies are applied^22^.

Despite numerous benefits, remote assessment is not without its limitations. First, it can be challenging to achieve the same level of standardization remotely as in traditional lab settings^23,24^ because researchers have less control over the testing environment (e.g., distraction). Second, depending on the level of monitoring, participants may engage in dishonest practices, such as taking notes, capturing screenshots, or seeking assistance from third parties. Third, if the remote assessments are administered on participants’ own devices, the assessment can be interrupted by phone calls, notifications, or text messages. Fourth, additional challenges to data safety and transfer exist, especially when Personally Identifiable Information (PII) is involved^25,26^.

The COVID-19 pandemic has also increased interest in the feasibility of remote cognitive assessment in pediatric populations^27,28^. A majority of the feasibility studies focused on the psychometric equivalency between remote and in-person cognitive assessments by using either intelligence scales such as the Wechsler Intelligence Scale for Children, Fifth Edition^29^ or other cognitive tests^27,28,30,31^. Additionally, some recent studies investigated remote cognitive assessment in special populations. In children with specific learning disabilities, for example, remote and in-person assessments of learning skills revealed similar results^32^. Likewise, remote administration of processing speed measures was feasible in children with chronic medical conditions^33^indicating its potential for broader use in clinical practice.

In the current pilot study, we investigate the equivalency of in-person and remote cognitive assessments in healthy children by examining a newly developed application - the *NIH Toolbox Participant/Examiner (NIHTB-P/E) App*, which leverages the *NIH Toolbox for Assessment of Neurological and Behavioral Function Cognition Battery* (NIHTB-CB; www.nihtoolbox.org*).* The NIHTB-CB is a well-established iPad-based measurement system currently deployed in over 1,100 institutions across the world^34–38^. It is used in several large-scale longitudinal studies such as Environmental influences on Child Health Outcomes (ECHO)^39^ and HEALthy Brain and Child Development (HBCD)^40^ is further used in clinical samples, including children with congenital heart defects (CHD)^41^ and those with Pompe disease (PD)^42^. Therefore, providing the remote option of the NIHTB-CB is an important contribution to the field by expanding accessibility, reducing barriers to participation, and enabling more frequent and flexible monitoring of cognitive development in pediatric populations.

**Fig. 1:**
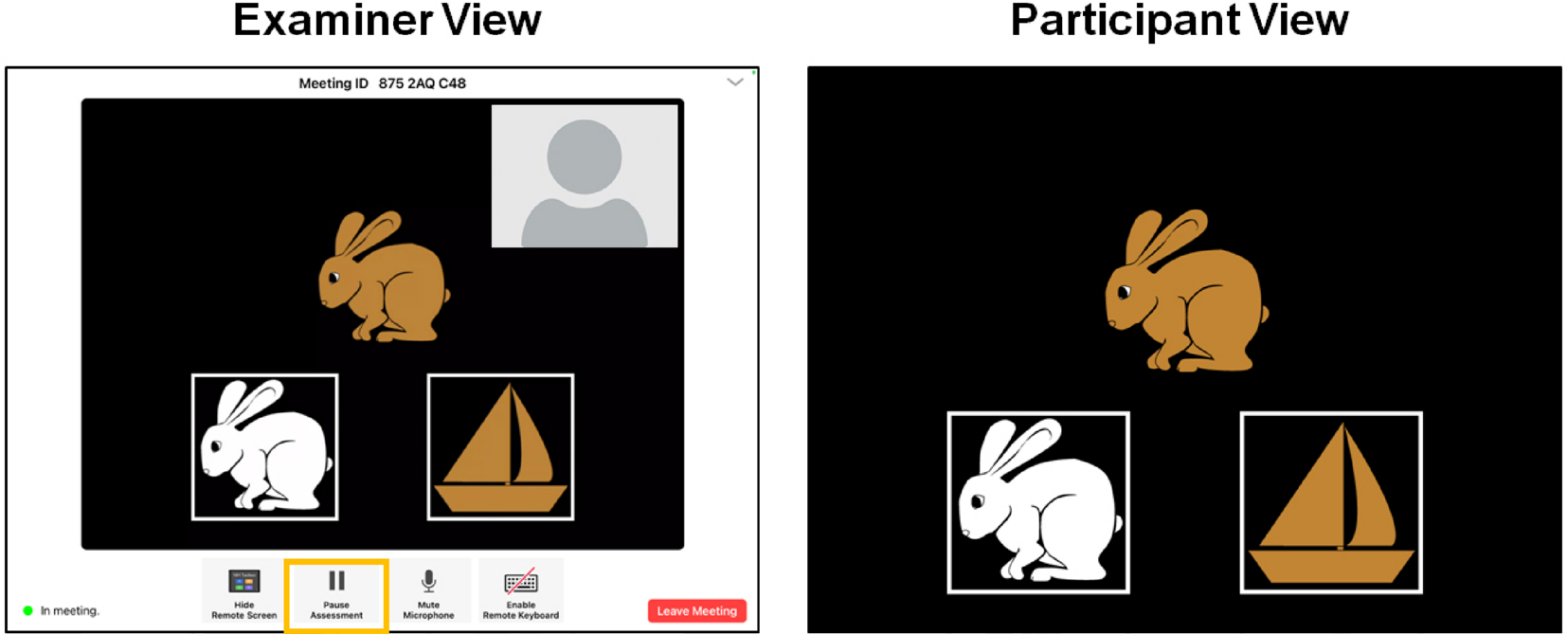
The NIH Toolbox Participant/Examiner App during a live session (DCCS Test).

**Fig. 2:**
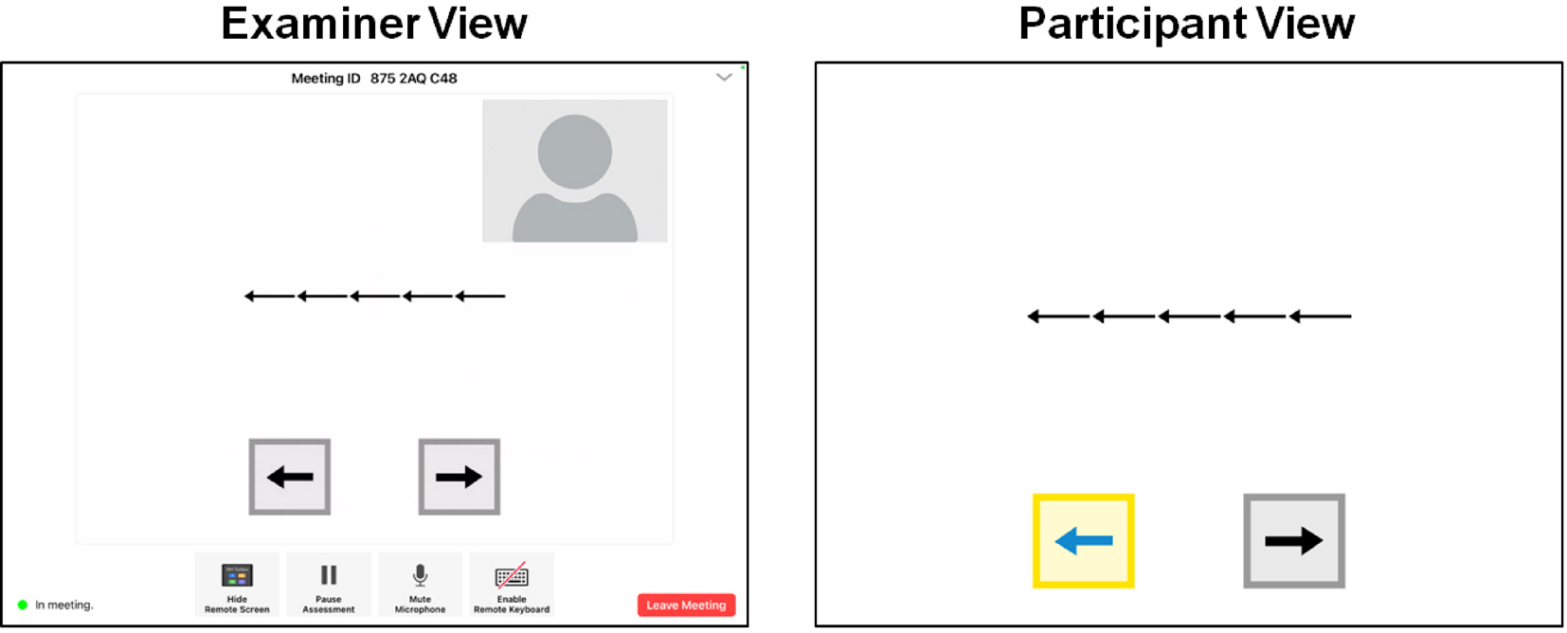
The NIH Toolbox Participant/Examiner App during a live session (Flanker Test).

Tests within the *NIH Toolbox Cognition Battery (NIHTB-CB)* span a diverse array of cognitive domains, including *working memory*,* processing speed*,* language*,* attention*,* executive functioning*, and *episodic memory* (see Table 1 for constructs, their definitions, corresponding tests, age ranges, and test durations), These tests have been proven useful for predicting cognitive performance across diverse childhood samples^43–46^. The NIHTB-CB measures were designed to be interactive, engaging, and developmentally appropriate and have previously been shown to be reliable and valid compared to similar gold-standard assessments in this age range^45,47^. NIHTB-CB measures have typically been administered in-person; however, they can now be administered remotely via the newly developed NIHTB-P/E app. This app is an iPad-to-iPad assessment system allowing for testing when the examiner and participant are in different locations. Critically, it includes a built-in bi-directional video-conferencing feature (see Figs. 1 and 2) that allows the administration to be experimenter-guided and fully monitored. This supervised remote cognitive assessment is similar to in-person testing due to the real-time interactions between the examiner and the participant through videoconferencing^48^.

**Table 1:** *Constructs measured in the NIH toolbox cognition battery and the NIH toolbox participant/examiner app together with their definitions*,* corresponding tests*,* age ranges*,* and test durations*.

Test	Construct	Definition	Age range	Duration (minutes)
List Sorting Working Memory Test	Working memory	The ability to retain and manipulate information in a temporary storage system	7–85	7
Pattern Comparison Processing Speed Test	Processing speed	The amount of time it takes to process a specific amount of information or the amount of information that can be processed within a specified timeframe.	7–85	3
Oral Reading Recognition Test	Language - Oral reading	Language is a system of symbols such as words that can be used for communication. Reading is the ability to pronounce these symbols.	7–85	3
Picture Vocabulary Test	Language - Vocabulary	One’s knowledge of the set of words in a specific language.	3–85	4
Flanker Inhibitory Control and Attention Test	Attention	The ability to allocate limited resources to deal with the abundant information in the environment.	3–85	4
Dimensional Change Card Sort Test Flanker Inhibitory Control and Attention Test	Executive function	A set of cognitive processes that enable individuals to plan, organize, monitor, and regulate behavior.	3–85	4
Picture Sequence Memory Test	Episodic Memory	The ability to acquire, store and retrieve new information and experiences learned within a specific context and encoded with time-specific information.	3–85	7

In this pilot study, we tested the equivalency of in-person cognitive assessment by using the NIHTB-CB and remote cognitive assessment via the NIHTB-P/E app. Children ages 7–17 completed the NIHTB-CB tests on an iPad, guided and monitored by a trained examiner using the NIHTB-P/E app. With the support of the bi-directional communication system, we expected no significant differences in test scores between remote cognitive assessment at home by using the NIHTB-P/E app and in-person assessment at the study site utilizing the NIHTB-CB.

## Method

### Participants

Child-caregiver dyads were recruited across six study sites throughout the United States (i.e., Orlando, FL; Houston, TX; Nashville, TN; Atlanta, GA; Baltimore, MD; and Dallas, TX) as part of a larger study^49^ with specific age, gender, race/education, and mother education targets to ensure demographic diversity (e.g., maximum 60% of each sex, at least 20% of mothers/caregivers with less than a college degree). Participants were screened by a market panel research company based on predetermined inclusion and exclusion criteria. Specifically, the inclusion criteria were: children aged 7–17 years, fluency in English, self-reported adequate internet access, and caregiver willingness to assist with the remote setup. Exclusion criteria, on the other hand, included a current positive COVID-19 test in the child, limited English proficiency in either the caregiver or the child, or a lack of access to an iPad-compatible internet connection. Children with physical impairments that would interfere with the ability to interact with the iPad (e.g., limited upper limb mobility preventing touchscreen use) were not included in this pilot study. A total of 58 child participants between 7 and 17 years old were recruited (48.3% female; *M*_*age*_ = 11.88, *SD*_*age*_ = 3.31). Of these participants, 47 (51.1% female; *M*_*age*_ = 12.26, *SD*_*age*_ = 3.23) completed both the in-person and remote cognitive batteries a few days apart (M = 3.15; *SD* = 3.06). While a small number of participants did not complete both sessions, there was no evidence of differential dropout by age group, sex, or mode of test administration. Demographic characteristics of the final sample are displayed in Table 2. Caregivers signed informed consent forms and received $225 for participating in both remote and in-person assessments. Their travel expenses were reimbursed for in-person site visits. The study protocol was approved by the WIRB-Copernicus Group (WCG) Institutional Review Board (IRB Approval #20231258). In addition, the study was conducted in accordance with the Declaration of Helsinki and applicable institutional/national ethical guidelines. Finally, written informed consent was obtained from all participants or their legally authorized representatives prior to study enrollment.

**Table 2:** Sample characteristics.

Characteristic	*n*	%
**Sex**		
Male	23	48.9
Female	24	51.1
**Age**		
7–12 years	24	51.1
13–17 years	23	48.9
**Race**		
White	27	57.4
Black or African American	18	38.3
Other	2	4.3
**Ethnicity**		
Not Hispanic or Latino	46	97.9
Hispanic or Latino	1	2.1
Total	47	100

## Measures

### The NIH toolbox cognition battery (NIHTB-CB)

*The NIH Toolbox for Assessment of Neurological and Behavioral Function* (NIHTB; www.nihtoolbox.org*)* is a comprehensive set of computerized measures with four batteries: cognition, emotion, motor, and sensation^37,50^. NIHTB is designed for use across the lifespan (i.e., ages 3 to 85) and has been reported to be a valid and reliable tool in different age groups and populations ranging from healthy adults to patients with neurological disorders^50–52^. The *NIHTB-CB* is designed to measure a broad range of cognitive abilities, including *attention*,* episodic memory*,* language (i.e.*,* oral reading* and *vocabulary)*,* working memory*,* executive function*, and *processing speed*. All tests in the NIHTB-CB are psychometrically validated and normed^50^. Each cognitive test in the battery is further described individually below.

## The NIH toolbox participant/examiner app (NIHTB-P/E)

*NIH Toolbox Participant/Examiner App* (NIHTB-P/E) is a newly developed iPad-to-iPad assessment system that allows for remote cognitive testing when the examiner and participant are in different locations. The NIHTB-P/E app leverages the NIHTB described above. The NIHTB-P/E app was designed for monitored, experimenter-guided assessment and, as such, includes a built-in bi-directional video-conferencing feature. The examiner can observe the participant completing the assessment at all times and has full control over the assessment, including pausing the assessment, terminating the assessment, and moving to a new measure. In turn, the participant is able to complete all measures directly on the iPad in front of them and, if necessary, can communicate with the examiner. Figures 1 and 2 show screenshots of the NIHTB-P/E app, captured during a sample testing session for the Dimensional Change Card Sort Test and the Flanker Inhibitory Control and Attention Test, respectively.

The NIHTB-P/E was designed to be adaptable to various iPad models, and as such, testing stimuli are fixed to the stimuli size of the NIHTB-CB, regardless of iPad screen size of either the examiner or participant. Additionally, scores are recorded and calculated locally on the participant’s device after each item (e.g., in a computer adaptive paradigm), maximizing data capture. The NIHTB-CB and NIHTB-P/E app offer parallel versions of standardized normed measures, with the only difference being the remote functionality of the latter system.

## Cognitive tests in the NIHTB-CB and the NIHTB-P/E app

**NIH Toolbox Dimensional Change Card Sort Test (DCCS).** NIH Toolbox Dimensional Change Card Sort Test^53^ measures cognitive flexibility, which is the ability to adaptively shift between sorting rules for identical stimuli. The original version of DCCS was developed by Zelazo and colleagues^54^ for the first version of the NIHTB-CB. This test is a measure of *fluid ability*, which is the capacity to acquire new knowledge and to adapt to unfamiliar circumstances. In DCCS, participants are shown two images side by side at the bottom of the screen. In each trial, participants see a cue word - either “shape” or “color” - at the center of the screen, followed by a bivalent target image, which participants sort based on the cued dimension. The sorting rule alternates between “color” and “shape” in a pre-determined order that appears to be pseudo-random. Participants respond by tapping one of the two visual images based on the dimension specified by the presented cue word (see Fig. 1). Scoring is based on both accuracy and reaction time.

**NIH Toolbox Flanker Inhibitory Control and Attention Test (Flanker).** The NIH Toolbox Flanker Inhibitory Control and Attention Test (Flanker) is a version of the Eriksen Flanker Task^55^ designed to measure attention and inhibitory control^36,53^. Like the DCCS test, Flanker is also considered a measure of *fluid ability*. In this test, each trial starts with a fixation star in the center of the screen, followed by a blank screen. Next, a row of five stimuli (fish or arrows) appears, pointing left or right (see Fig. 2). Participants are instructed to tap one of two buttons on the bottom of the screen that matches the target stimulus’s direction (the middle fish or arrow). Scoring is based on both accuracy and reaction time.

**NIH Toolbox List Sorting Working Memory Test (LSWM).** The NIH Toolbox List Sorting Working Memory Test (LSWM) is a sequencing task developed to measure working memory^56^. In this test, which is a measure of *fluid ability*, participants engage in immediate recall and sequencing of different stimuli presented visually and orally. A set of pictures of different animals and foods are presented with an accompanying audio recording and written text (e.g., dog, apple). The participants are then asked to say the items back in size order (smallest to largest) in two formats, first within a single dimension (either animals or foods, called 1-List) and then in two dimensions (foods, then animals, called 2-List). Scoring is based on the total number of items correct.

**NIH Toolbox Pattern Comparison Processing Speed Test (PCPS).** The NIH Toolbox Pattern Comparison Processing Speed Test (PCPS) is a measure of *fluid ability* and developed to assess the speed of processing. It measures how accurately participants can decide whether two side-by-side pictures are the same^51^. When the presented patterns are not identical, they vary on one of three dimensions (i.e., color, quantity, and presence/absence of an image or image component). Participants respond to whether or not the patterns are identical by pressing a “yes” or “no” button. Scoring is based on the total number of items correct.

**NIH Toolbox Picture Sequence Memory Test (PSM).** The NIH Toolbox Picture Sequence Memory Test (PSM) measures episodic memory by asking participants to recall the order of thematically related pictures of objects and activities^57,58^. It is also considered as a measure of *fluid ability*. At the beginning of each trial, a fixed spatial order of pictures is displayed in the center of the screen, with an auditory description of an activity that can be described with all the pictures. Immediately following the presentation of a sequence of pictures, the participants are instructed to re-order the stimuli from memory. Participants are asked to recall each sequence twice. The number of presented pictures in a sequence varies between 6 and 18 depending on the age of the participant. Participants are given credit for each adjacent pair of pictures they correctly place (i.e., if pictures in locations 7 and 8 are placed in that order and adjacent to each other anywhere, such as slots 1 and 2, one point is awarded), up to the maximum value for the sequence, which is one less than the sequence length. Scoring is based on an IRT-based score where the number of correct adjacent pairs is transformed into a latent (theta) score, which is then scaled to a normed score.

**NIH Toolbox Oral Reading Recognition Test (OR).** The NIH Toolbox Oral Reading Recognition Test (OR) employs a Computer Adaptive Testing^59,60^ methodology, requiring active administrator involvement for scoring^60,61^. It measures *crystallized abilities*, which develop with age and education and increase during childhood before becoming stable in adulthood. The examiner first identifies the educational level of the participant in order to set the appropriate starting point. Respondents are then provided with a word on the screen. The difficulty level of the words is set according to the participant’s age and adaptively increases or decreases in difficulty based on the participant’s performance. Participants are asked to pronounce each word to the best of their ability. Examiners are trained with audio recordings for the word list and a printed pronunciation guide before administering the test. The trained examiner scores the participants’ responses as either “correct” or “incorrect” based on pronunciation accuracy. Pronunciations that did not match the respelling pronunciation guide were evaluated as incorrect. Scoring is based on a combination of correct responses and the difficulty of each item, and a latent (theta) score is produced that is then scaled to normed scores.

**NIH Toolbox Picture Vocabulary Test (PVT).** The NIH Toolbox Picture Vocabulary Test (PVT) assesses general vocabulary knowledge^60^ using CAT. This test is a measure of *crystallized abilities*. The examiner first identifies the educational level of the participant in order to set the appropriate starting point. During the test, the participant is presented with four photographic images on the screen and an audio recording that matches one of the four images. Participants are provided as much time as they need to respond and are asked to select the picture that matches most closely based on the recording. The difficulty level of the words is set according to the participant’s age and adaptively increases or decreases in difficulty based on the participant’s performance. Scoring is based on a combination of correct responses and the difficulty of each item, and a latent (theta) score is produced that is then scaled to normed scores.

### Procedure

Prior to data collection, examiners were trained and certified to administer both the in-person and remote versions of the NIHTB-CB. The order of remote and in-person cognitive assessment sessions was counterbalanced, with half of the participants (randomly selected) first completing the assessments remotely and the other half completing them in person.

For the remote assessments participants were shipped a study kit that included a study iPad pre-loaded with the NIHTB-P/E app, an iPad charger, printed instructions for setup and use, and paper copies of data collection forms to be completed during the remote session. Caregivers were provided with step-by-step instructions on how to enter a meeting code on the NIHTB-P/E app that would connect them to the examiner. Once connected with the examiner on the app, caregivers followed the examiner’s live instructions through the app’s communication system to assist with the final setup process (e.g., adjusting the volume on the iPad).

The setup process on the participant’s end typically took less than five minutes, not including the time to charge the iPad, which families were instructed to do in advance. Once the set-up process was complete, caregivers were instructed not to assist their child with any tests. However, they were allowed to help with technical difficulties, such as connection issues or iPad malfunctions. The examiner could note any deviations from the administration, though there were none recorded regarding the administration of the current reported study. Caregivers were also given the examiner’s contact information in case the examiner was disconnected during the test and needed to rejoin the app. After completing the assessments, participants could return the iPad either in person at the study site or by using a prepaid return shipping label provided in the kit.

## Statistical analyses

### Individual and composite test scores

Individual test scores were obtained from each of the seven tests in the NIHTB-CB. Composite scores were derived from a specific combination of individual test scores, resulting in three categories: *fluid composite (FC)*,* crystallized composite (CC)*, and *total composite (TC)* test scores^34,62^. Specifically, the FC test score includes Flanker, Dimensional Change Card Sort, Picture Sequence Memory, List Sorting, and Pattern Comparison Tests while the CC test score includes the Picture Vocabulary and Reading Tests. These composite scores were calculated by averaging the standard scores of the individual tests. Finally, the TC test score is the average of the FC and CC test scores. These composites were empirically derived in prior validation studies of the NIH Toolbox Cognitive Battery and have been used in previous research involving children and adolescents^63^.

### Age-corrected standard scores and uncorrected standard scores

For each test, we used two types of test scores: *age-corrected standard scores* and *uncorrected standard scores.* Age-corrected standard scores compare each participant’s score to those in the original NIHTB norming study of nationally representative individuals of the same age^36^. A score of 100 indicated performance at the national average for the participant’s age with an SD of 15. Uncorrected standard scores also use a standard score metric (normative mean = 100 and SD = 15), comparing the performance of the test-taker to those in the entire NIHTB normative sample, regardless of age or any other variable. In the present study, all analyses involving test scores were conducted separately for age-corrected and uncorrected standard scores for comparison purposes. Results based on uncorrected standard scores are presented in Table S1 and Table S2 in the supplementary materials.

Finally, analyses involving participants’ age used two age bands: 7- to 12-years and 13- to 17-years, consistent with previous research using the NIHTB-CB in child samples^36,45,64^. We also conducted the analyses by including age as a continuous covariate and obtained consistent results, indicating that our findings are robust regardless of how age is included in the analyses.

### Group comparisons

Differences between remote and in-person cognitive assessment scores were compared by conducting a series of Repeated Measures ANOVAs. The *mode of administration* (remote vs. in-person) was the within-subjects while *age group* (7-to-12 vs. 13-to-17 years old) and *administration order* (remote first vs. in-person first) were between-subjects factors. Another series of Repeated Measures ANOVAs examined the within-subjects effect of *mode of administration* (remote vs. in-person) controlling for *age group* (7-to-12 vs. 13-to-17 years old) and *administration order* (remote first vs. in-person first), which were the between-subjects factors on test time in minutes. Bonferroni corrections^65^ were applied to adjust for multiple comparisons, with an alpha level of 0.017 for analyses involving the three composite test scores and 0.007 for analyses involving the seven individual test scores.

### Analysis of overlap

To assess the similarity between the empirical distributions of each measure and composite when compared by administration mode (e.g., the percentage overlap in distribution between remote and in-person Pattern Comparison Processing Speed Test), we calculated the overlap between their respective kernel density estimates^66^. This analysis has been used in many contexts in many fields^67,68^is efficient to calculate, makes no assumptions of normality, and is straightforward to interpret. This analysis was done using the ‘overlapping’ package in R 4.2.2^69,70^.

## Results

### Mode of administration by age group and administration order

Analyses on participants’ individual test scores revealed no significant differences between remote and in-person cognitive assessments (see Table 4). Age group and administration order had no significant effects on performance for individual test scores (see Table 4). However, there was a significant interaction between the mode of administration and the first mode for two of the individual tests: Pattern Comparison Processing Speed Test and Picture Sequence Memory. To be more specific, test scores for remote cognitive assessments were lowest for these two tests when the remote assessment was administered first, whereas they were highest when the in-person cognitive assessment was administered first (see Table 3). As seen in Table 4, no other significant interaction between the mode of administration and the first mode was observed for the remaining individual test scores. Finally, the three-way interaction between mode of administration, age group, and first mode was not significant for individual test scores (see Table 4). Results of the separate analyses for composite test scores are provided in the Supplementary Materials. Specifically, Table S1 presents the means and standard deviations of age-corrected composites test scores by mode of administration, age and first mode and Table S2 presents mean square error (MSE), F and p values together with the effect sizes of the Repeated Measures ANOVAs. As seen in Table S2, results for composite scores generally followed the same pattern observed in individual test scores with significant Mode*First Mode interactions for both the fluid and total composites, suggesting higher remote scores when remote testing was the second administration (see Supplementary Table S1).

**Table 3:** *Means and standard deviations of age-corrected standard scores by mode of administration*,* age and first mode*.

	7–12 years old		13–17 years old		Remote first		In-person first	
Measure	M	SD	M	SD	M	SD	M	SD
**DCCS**								
Remote	95.00	15.05	98.22	16.64	95.61	13.57	98.44	19.69
In-person	96.75	14.38	95.87	17.15	96.87	14.38	95.25	18.26
**Flanker**								
Remote	92.65	9.76	91.78	18.00	91.48	13.85	93.69	15.32
In-person	98.58	11.95	86.61	15.51	92.90	15.35	92.38	11.57
**List Sort**								
Remote	100.33	17.01	103.43	12.64	102.60	15.61	101.44	14.06
In-person	95.21	12.58	102.17	10.43	100.16	11.94	95.63	11.87
**Pattern Comparison**								
Remote	97.29	29.59	110.09	22.30	97.00	25.74	116.25	24.78
In-person	104.38	21.39	120.74	21.02	116.87	22.99	103.69	19.50
**Picture Sequence Memory**								
Remote	104.38	17.49	101.30	19.49	98.00	15.54	112.31	20.16
In-person	109.00	18.05	105.74	17.23	109.45	18.81	103.44	14.48
**Oral Reading**								
Remote	101.42	19.79	100.83	17.40	101.58	18.87	100.25	18.21
In-person	101.46	19.28	99.96	15.14	101.39	17.17	99.44	17.77
**Picture Vocabulary**								
Remote	102.46	13.67	99.39	13.83	101.23	13.97	100.44	13.56
In-person	99.25	12.79	97.74	11.44	98.48	11.97	98.56	12.57

**Table 4:** *Comparison of age-corrected standard scores by mode of administration*,* age*,* and first mode*.

Score	MSE	F	*p*	partial ηp²
**DCCS**				
Mode	60.14	0.818	0.371	0.019
Age	6.11	0.014	0.907	0.000
First mode	5.03	0.011	0.916	0.000
Mode*Age	218.35	2.969	0.092	0.065
First mode*Age	94.17	0.213	0.647	0.005
Mode*First mode	207.77	2.826	0.100	0.062
Mode*Age*First mode	54.65	0.743	0.393	0.017
**Flanker**				
Mode	4.05	0.045	0.833	0.001
Age	1,352.82	4.346	0.043	0.092
First mode	71.57	0.230	0.634	0.005
Mode*Age	701.35	7.807	0.008	0.154
First mode*Age	439.05	1.411	0.241	0.032
Mode*First mode	163.99	1.825	0.184	0.041
Mode*Age*First mode	4.75	0.053	0.819	0.001
**List Sort**				
Mode	233.76	2.344	0.133	0.052
Age	743.14	2.778	0.103	0.061
First mode	3.93	0.015	0.904	0.000
Mode*Age	59.39	0.595	0.445	0.014
First mode*Age	326.36	1.220	0.276	0.028
Mode*First mode	38.05	0.381	0.540	0.009
Mode*Age*First mode	6.00	0.060	0.807	0.001
**Pattern Comparison**				
Mode	399.72	2.495	0.122	0.055
Age	2,979.41	3.606	0.064	0.077
First mode	533.20	0.645	0.426	0.015
Mode*Age	12.93	0.081	0.778	0.002
First mode*Age	1,959.84	2.372	0.131	0.052
Mode*First mode	4,853.31	30.299	< 0.001	0.413
Mode*Age*First mode	280.72	1.753	0.193	0.039
**Picture Sequence Memory**				
Mode	0.06	0.000	0.986	0.000
Age	0.32	0.001	0.978	0.000
First mode	424.21	1.003	0.322	0.023
Mode*Age	282.31	1.554	0.219	0.035
First mode*Age	644.34	1.524	0.224	0.034
Mode*First mode	2,510.62	13.817	< 0.001	0.243
Mode*Age*First mode	177.15	0.975	0.329	0.022
**Oral Reading**				
Mode	15.00	0.263	0.611	0.006
Age	0.82	0.001	0.971	0.000
First mode	22.24	0.036	0.850	0.001
Mode*Age	17.58	0.308	0.582	0.007
First mode*Age	412.76	0.675	0.416	0.015
Mode*First mode	8.78	0.154	0.697	0.004
Mode*Age*First mode	21.34	0.374	0.544	0.009
**Picture Vocabulary**				
Mode	71.88	1.875	0.178	0.042
Age	276.83	0.900	0.348	0.020
First mode	57.88	0.188	0.667	0.004
Mode*Age	29.80	0.778	0.383	0.018
First mode*Age	224.27	0.729	0.398	0.017
Mode*First mode	13.96	0.364	0.549	0.008
Mode*Age*First mode	13.17	0.344	0.561	0.008

Note.Effect sizes reported are partial eta squared (partial η²). Benchmarks for interpreting partial η² are: small = 0.0099, medium = 0.0588, and large = 0.1379 based on Richardson (2011)^80^. *MSE*: Mean Square Error.

### Test duration by mode of administration, age group, and administration order

Results indicated a significant main effect of mode of administration on test duration for Dimensional Change Card Sort Test, List Sorting Working Memory, and Picture Sequence Memory tests: Participants took longer to complete these tests in remote administration compared to in-person administration (see Tables 5 and 6). Mode of administration had no effect on the remaining tests of Flanker, Pattern Comparison Processing Speed Test, Oral Reading Recognition, and Picture Vocabulary (see Table 6). Age group had a main effect on test duration for the Pattern Comparison Processing Speed Test, with younger participants completing the test faster than their older counterparts (see Tables 5 and 6). The interaction between the mode of administration and administration order was significant for Pattern Comparison Processing Speed Test and Picture Vocabulary tests (see Table 6). For the Pattern Comparison Processing Speed Test, children completed the test faster in the first administration. More specifically, in-person testing had shorter duration than remote testing when it was conducted first while remote assessment was shorter than in-person one when it was administered first. For the Picture Vocabulary test, duration was the longest when the cognitive assessment was completed remotely as the first measurement. However, remote Picture Vocabulary testing displayed the shortest duration when the first administration mode was in-person. No other significant effects or interactions were observed (see Table 6).

**Table 5:** Means and standard deviations of test durations in minutes by mode of administration and age.

	Remote		In-person	
Test	M	SD	M	SD
**DCCS**				
7–12 years old	5.68	0.98	5.24	0.79
13–17 years old	5.26	0.39	4.92	0.32
Total	5.48	0.77	5.08	0.62
**Flanker**				
7–12 years old	4.51	1.68	3.96	1.06
13–17 years old	4.24	2.01	3.37	0.28
Total	4.38	1.87	3.67	0.83
**List Sort**				
7–12 years old	9.23	2.99	7.16	1.51
13–17 years old	8.71	2.28	7.32	2.34
Total	8.98	2.65	7.24	1.94
**Pattern Comparison**				
7–12 years old	1.65	0.06	1.67	0.04
13–17 years old	1.71	0.05	1.73	0.04
Total	1.68	0.06	1.70	0.05
**Picture Sequence Memory**				
7–12 years old	8.31	2.16	7.46	1.66
13–17 years old	8.17	2.37	6.45	0.79
Total	8.24	2.24	6.97	1.39
**Oral Reading**				
7–12 years old	1.83	0.74	1.67	0.81
13–17 years old	2.17	1.70	1.42	0.53
Total	2.00	1.30	1.55	0.69
**Picture Vocabulary**				
7–12 years old	2.27	0.98	2.05	0.55
13–17 years old	2.13	1.29	1.74	0.66
Total	2.20	1.13	1.90	0.62

**Table 6:** *Test durations by mode of administration*,* age*,* and first mode*.

Test	MSE	F	*p*	ηp²
**DCCS**				
Mode	2.570	40.023	< 0.001	0.482
Age	1.283	1.783	0.189	0.040
First mode	1.264	1.757	0.192	0.039
Mode*Age	0.081	1.262	0.268	0.028
First mode*Age	5.252	7.299	0.010	0.145
Mode*First mode	0.163	2.531	0.119	0.056
Mode*Age*First mode	0.016	0.254	0.617	0.006
**Flanker**				
Mode	5.474	4.298	0.044	0.091
Age	3.916	1.308	0.259	0.030
First mode	1.318	0.44	0.511	0.01
Mode*Age	0.004	0.003	0.957	0.000
First mode*Age	0.246	0.082	0.776	0.002
Mode*First mode	2.804	2.202	0.145	0.049
Mode*Age*First mode	0.638	0.501	0.483	0.012
**List Sort**				
Mode	51.392	15.184	< 0.001	0.261
Age	0.637	0.088	0.769	0.002
First mode	0.381	0.052	0.820	0.001
Mode*Age	2.028	0.599	0.443	0.014
First mode*Age	19.639	2.702	0.108	0.059
Mode*First mode	7.145	2.111	0.153	0.047
Mode*Age*First mode	4.662	1.377	0.247	0.031
**Pattern Comparison**				
Mode	0.002	2.717	0.107	0.059
Age	0.056	16.762	< 0.001	0.280
First mode	0.003	0.969	0.330	0.022
Mode*Age	2.203	0.032	0.858	0.001
First mode*Age	0.004	1.310	0.259	0.030
Mode*First mode	0.017	25.629	< 0.001	0.373
Mode*Age*First mode	0.000	0.660	0.421	0.015
**Picture Sequence Memory**				
Mode	19.068	8.695	0.005	0.168
Age	7.565	1.802	0.187	0.040
First mode	8.755	2.085	0.156	0.046
Mode*Age	1.071	0.489	0.488	0.011
First mode*Age	2.026	0.482	0.491	0.011
Mode*First mode	16.853	7.685	0.008	0.152
Mode*Age*First mode	0.222	0.101	0.752	0.002
**Oral Reading**				
Mode	1.940	2.740	0.105	0.060
Age	0.031	0.021	0.886	0.000
First mode	0.395	0.267	0.608	0.006
Mode*Age	0.671	0.948	0.336	0.022
First mode*Age	0.482	0.326	0.571	0.008
Mode*First mode	2.794	3.946	0.053	0.084
Mode*Age*First mode	0.049	0.070	0.793	0.002
**Picture Vocabulary**				
Mode	0.648	1.896	0.176	0.042
Age	1.449	1.158	0.288	0.026
First mode	2.026	1.618	0.210	0.036
Mode*Age	0.000	0.001	0.971	0.000
First mode*Age	0.148	0.119	0.732	0.003
Mode*First mode	3.527	10.313	0.003	0.193
Mode*Age*First mode	0.117	0.341	0.562	0.008

Note. Effect sizes reported are partial eta squared (partial η²). Benchmarks for interpreting partial η² are: small = 0.0099, medium = 0.0588, and large = 0.1379 (Richardson, 2011)^80^. *MSE*: Mean Square Error.

### Analysis of overlap

**Fig. 3:**
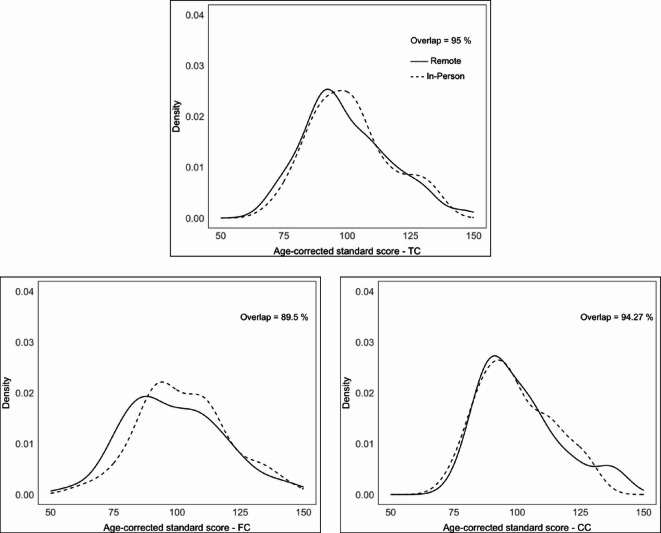
Density plots with percentage overlap for age-corrected composite test scores.

The percentage overlap between remote and in-person distributions of each NIHTB-CB test ranged between 96.61% and 84.21%, indicating the highest overlap for the Flanker test and the lowest for the List Sorting Working Memory test. The mean percentage overlap for all seven tests was 90.72%. The percentage overlap for the fluid, crystallized, and total composite scores was 89.5%, 94.27%, and 95%, respectively. Density plots with the percentage overlap between in-person and remote assessments are presented in Fig. 3 for composite test scores. For individual test scores, density with the percentage overlap between in-person and remote assessments are displayed in Figure S1 in the supplementary materials.

## Discussion

Results of this pilot study show equivalency between in-person and remote test scores, indicating that the NIHTB-P/E app is a feasible option for remote cognitive assessment in children aged 7–17. Introducing standardized remote cognitive assessment methods is critical, as remote assessment has endured in the post-pandemic era^71,72^ and offers a range of potential benefits, including increased diversity and representativeness of research subjects. Increasing sample diversity is essential for research with children as developmental processes can vary depending on geographical location, ethnicity, and socioeconomic status^73–75^. Through remote assessment, children who would otherwise be excluded from studies, such as those living in rural areas, face lower barriers to participation, all of which can help improve the ecological validity of research studies. In addition to these benefits, remote tools like the NIHTB-P/E app can enhance the feasibility of decentralized clinical trials (DCTs)^76,77^ by reducing reliance on in-person site visits. Thus, the remote administration of the NIHTB-CB will support future studies including the decentralized ones.

The lack of significant differences between in-person and remote scores held across age groups (7–12 and 13–17 years old) and, for the most part, regardless of the order in which the testing modes were administered. Indeed, for five out of the seven tests, no significant differences were observed in scores across the two formats. However, the interaction between the mode of administration and the order of remote versus in-person assessments revealed practice effects for two individual tests: the Pattern Comparison Processing Speed and Picture Sequence Memory tests. More specifically, taking these tests in-person first led to higher scores on the second (remote) session. Interestingly, when the remote assessment was administered first, we did not see an increase in performance in the second (in-person) session. It is important to note that practice effects on these tests may not be specific to this context since previous studies have also reported practice effects for both Pattern Comparison Processing Speed^51^ and Picture Sequence Memory^58^. These effects may be related to familiarity with stimuli and may have more to do with the relatively short time between the remote and in-person administrations in the current study. Overall, current findings indicate that the NIHTB-P/E app, in general, offers a way to reliably assess cognitive abilities in decentralized protocols. It is important to note that this analysis was focused on group-level equivalency between in-person and remote testing, often used in cross-sectional research designs, for example, rather than individual-level measurement agreement. As such, while preliminary findings of our pilot study support the comparability of in-person and remote testing at the group level, future research is needed to investigate whether individuals obtain consistent scores across administrations.

Additionally, we found that test duration was impacted by the mode of administration, with participants taking significantly longer to complete specific tests (i.e., Dimensional Change Card Sort, List Sorting Working Memory, and Picture Sequence Memory) remotely compared to in-person. Age differences affected only the Pattern Comparison Processing Speed test, which was completed faster by younger participants than older ones. The Picture Vocabulary test had the longest duration when administered remotely first and shortest when remote testing followed in-person administration. The Pattern Comparison Processing Speed test, on the other hand, had a shorter duration in the first administration than the second, independent of the mode of administration. These differences, however, had very small effect sizes. Overall, these findings suggest that both administration mode and order have nuanced effects on test duration for the Pattern Comparison Processing Speed and Picture Vocabulary tests, highlighting the importance of considering these factors when interpreting remote versus in-person testing results.

One limitation of the NIHTB-P/E app is that it requires sufficient internet bandwidth and the availability of required technological devices: in this case, an iPad. While these limitations may be an issue by introducing selection bias, systematically excluding individuals lacking access to internet services, technological devices, and technological literacy^78,79^we note that there are many solutions to overcome these challenges. For instance, researchers can send participants iPads with built-in internet service or connect users to locations with adequate internet access, such as libraries. Certainly, our study addressed this limitation by screening participants for internet access and providing iPads for home use. However, these requirements may present significant challenges for larger, multi-site, or national studies involving hundreds or thousands of participants.

Another limitation to note is that although our study demonstrated equivalency between the remotely applied NIHTB-P/E app and the in-person NIHTB-CB, the norm-referenced scores available for the NIHTB are based on data from in-person testing. This creates future opportunities to harmonize assessments across both modes of administration. Finally, the moderate sample size, consistent with the pilot nature of the study, limits the generalizability of the current findings and requires further research with larger samples. While the study provides important preliminary evidence supporting remote administration of the NIHTB-CB, the findings should be interpreted with caution. Larger studies are needed to confirm these results and explore variability across different subgroups and settings.

In conclusion, the NIHTB-P/E app provides a feasible and standardized method for remote administration of the NIHTB-CB to children in varied environments. Although this pilot study had a small sample size that may limit statistical power to detect subtle differences, the findingssuggest that scores obtained remotely are generally comparable to those from traditional in-person methods These preliminary results support the potential utility of the app for remote cognitive assessment with minimal impacts on test duration or performance. This pilot study supports the NIHTB-P/E app’s potential to expand the scope of cognitive assessment research, reducing participant burden.

## Supplementary Information

Below is the link to the electronic supplementary material.

Supplementary Material 1

## Data Availability

The data that support the findings of this study are available from the corresponding author (BE) upon reasonable request.
