# Supplementary material for: A pilot study of remote cognitive assessment in children using the NIH toolbox participant/examiner app
Source: Sci Rep. 2025 Oct 16;15:36204. doi: 10.1038/s41598-025-20256-7 (PMC12533012; doi:10.1038/s41598-025-20256-7)
Supplement: Supplementary file 1 — Supplementary Material 1 [file 41598_2025_20256_MOESM1_ESM.docx]

**Supplementary Materials**

**Table S1**

*Means and standard deviations of age-corrected standard scores by mode of administration, age and first mode*

|  | 7-12 years old | | | 13-17 years old | | | Remote first | | | In-person first | |
| --- | --- | --- | --- | --- | --- | --- | --- | --- | --- | --- | --- |
| Measure | M | SD | M | | SD | M | | SD | M | | SD |
| Fluid Composite |  |  |  | |  |  | |  |  | |  |
| Remote | 96.88 | 20.17 | 101.17 | | 17.50 | 94.26 | | 16.91 | 108.13 | | 19.51 |
| In-person | 101.58 | 17.48 | 103.39 | | 17.87 | 105.32 | | 18.87 | 96.94 | | 13.33 |
| Crystallized Composite |  |  |  | |  |  | |  |  | |  |
| Remote | 102.25 | 17.356 | 100.17 | | 15.5 | 101.71 | | 17.022 | 100.31 | | 15.387 |
| In-person | 100.42 | 15.399 | 98.57 | | 17.737 | 99.9 | | 15.23 | 98.75 | | 13.359 |
| Total Composite |  |  |  | |  |  | |  |  | |  |
| Remote | 99.17 | 18.09 | 100.57 | | 16.22 | 97.29 | | 17.00 | 104.81 | | 16.48 |
| In-person | 101.04 | 16.13 | 100.83 | | 16.17 | 102.84 | | 17.22 | 97.25 | | 12.94 |

**Table S2**

*Comparison of age-corrected standard scores by mode of administration, age, and first mode*

| Score | MSE | F | p | partial ηp² |
| --- | --- | --- | --- | --- |
| Fluid Composite |  |  |  |  |
| Mode | 2.36 | .029 | .865 | .001 |
| Age | 152.11 | .282 | .598 | .007 |
| First mode | 190.85 | .353 | .555 | .008 |
| Mode*Age | 303.46 | 3.730 | .060 | .080 |
| First mode*Age | 173.26 | .321 | .574 | .007 |
| Mode*First mode | 2,787.69 | 34.266 | <.001 | .443 |
| Mode*Age*First mode | 4.87 | .060 | .808 | .001 |
| Crystallized Composite |  |  |  |  |
| Mode | 55.15 | 2,319.000 | .135 | .051 |
| Age | 86.18 | .179 | .674 | .004 |
| First mode | 58.82 | .122 | .728 | .003 |
| Mode*Age | .20 | .008 | .927 | .000 |
| First mode*Age | 11.53 | .024 | .878 | .001 |
| Mode*First mode | .35 | .015 | .904 | .000 |
| Mode*Age*First mode | .32 | .013 | .908 | .000 |
| Total Composite |  |  |  |  |
| Mode | 28.85 | .875 | .355 | .020 |
| Age | 2.89 | .006 | .941 | .000 |
| First mode | 16.40 | .031 | .860 | .001 |
| Mode*Age | 117.89 | 3.576 | .065 | .077 |
| First mode*Age | 32.40 | .062 | .805 | .001 |
| Mode*First mode | 982.23 | 29.798 | <.001 | .409 |
| Mode*Age*First mode | .39 | .012 | .914 | .000 |

*Note.* Effect sizes reported are partial eta squared (partial η²). Benchmarks for interpreting partial η² are: small = .0099, medium = .0588, and large = .1379 based on Richardson (2011).^80^

MSE: Mean Square Error

**Figure S1**

*Density plots with percentage overlap for age-corrected individual test scores*

| 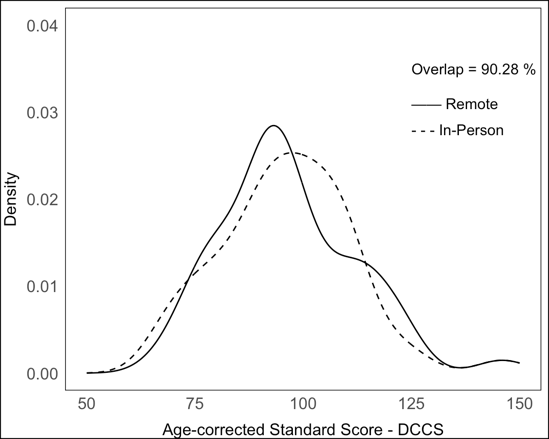 | |
| --- | --- |
| 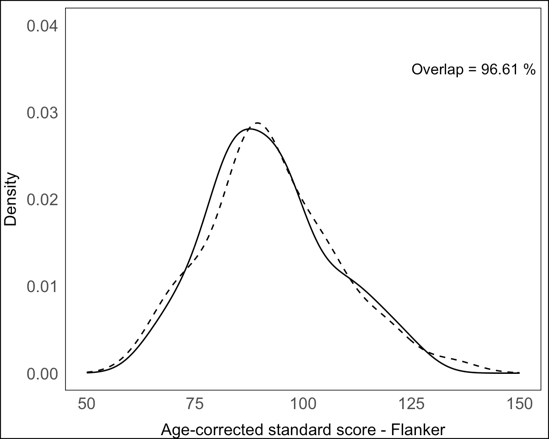 | 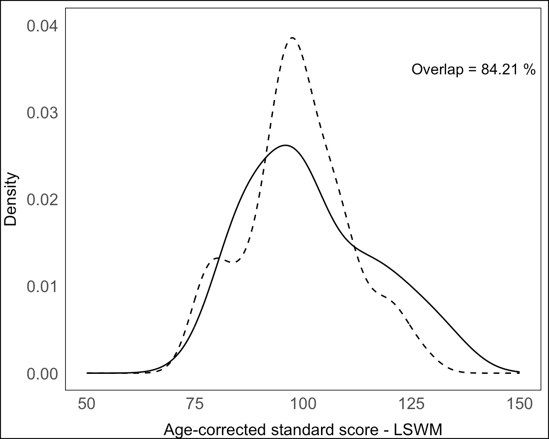 |
| 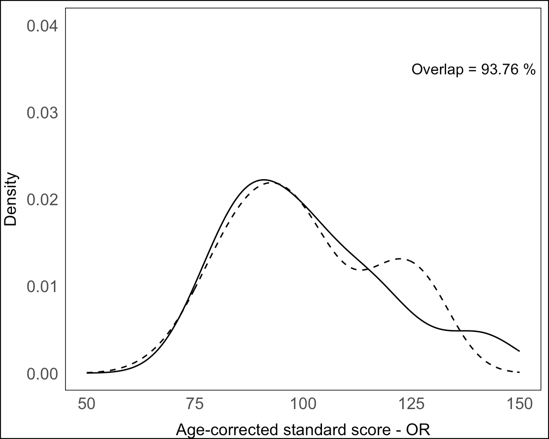 | 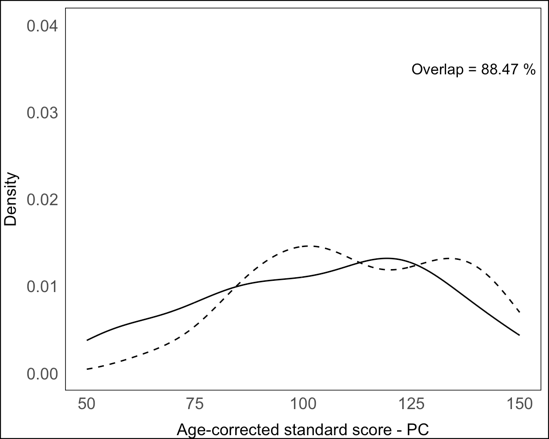 |
| 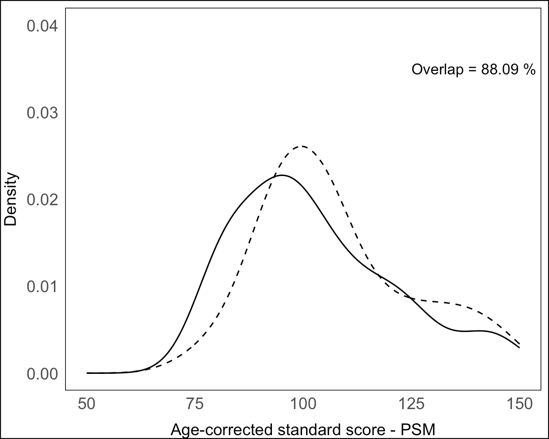 | 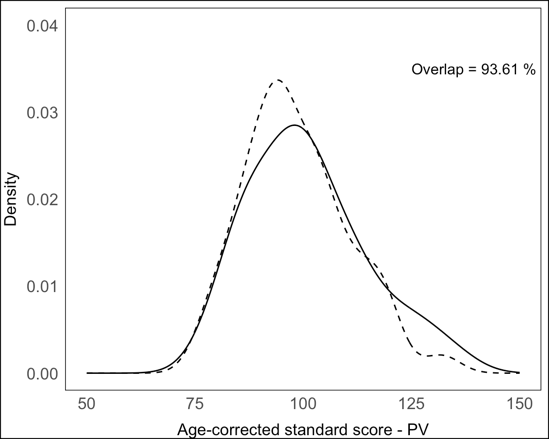 |
